# 3,4-Diphenyl-2,5-bis­(trimethyl­silyl)cyclo­penta­dienone

**DOI:** 10.1107/S1600536808032923

**Published:** 2008-10-18

**Authors:** Masaichi Saito, Toru Ito

**Affiliations:** a Department of Chemistry, Graduate School of Science and Engineering, Saitama University, Shimo-okubo, Saitama City, Saitama 338-8570, Japan

## Abstract

In the title compound, C_23_H_28_OSi_2_, the five-membered ring is essentially planar and the phenyl rings are oriented with respect to the mean plane of this ring by 56.01 (3) and 56.68 (4)°.

## Related literature

For a previous report of the synthesis of the title compound, see: Rajesh & Periasamy (1999 ▶). For related structures, see: Barnes *et al.* (1991 ▶); Ruffani *et al.* (2006 ▶).
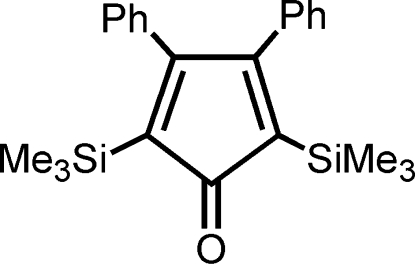


## Experimental

### 

#### Crystal data

C_23_H_28_OSi_2_
*M* *_r_* = 376.63
Orthorhombic, 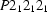

*a* = 9.8418 (6) Å
*b* = 11.8041 (7) Å
*c* = 19.0181 (12) Å
*V* = 2209.4 (2) Å^3^
*Z* = 4
Mo *K*α radiation
μ = 0.17 mm^−1^
*T* = 103 (3) K
0.50 × 0.40 × 0.40 mm

#### Data collection

Bruker SMART CCD area-detector diffractometer
Absorption correction: multi-scan (*SADABS*; Sheldrick, 1996 ▶) *T*_min_ = 0.922, *T*_max_ = 0.935
16200 measured reflections
5286 independent reflections
5181 reflections with *I* > 2σ(*I*)
*R*_int_ = 0.021

#### Refinement

*R*[*F* ^2^ > 2σ(*F* ^2^)] = 0.028
*wR*(*F* ^2^) = 0.075
*S* = 1.07
5286 reflections
241 parameters
H-atom parameters constrained
Δρ_max_ = 0.28 e Å^−3^
Δρ_min_ = −0.20 e Å^−3^
Absolute structure: Flack (1983 ▶), with 2292 Friedel pairs
Flack parameter: 0.01 (6)

### 

Data collection: *SMART* (Bruker, 2000 ▶); cell refinement: *SAINT* (Bruker, 2000 ▶); data reduction: *SAINT*; program(s) used to solve structure: *SHELXS97* (Sheldrick, 2008 ▶); program(s) used to refine structure: *SHELXL97* (Sheldrick, 2008 ▶); molecular graphics: *SHELXTL* (Sheldrick, 2008 ▶); software used to prepare material for publication: *SHELXTL*.

## Supplementary Material

Crystal structure: contains datablocks global, I. DOI: 10.1107/S1600536808032923/pv2107sup1.cif

Structure factors: contains datablocks I. DOI: 10.1107/S1600536808032923/pv2107Isup2.hkl

Additional supplementary materials:  crystallographic information; 3D view; checkCIF report

## Figures and Tables

**Fig. 1.**
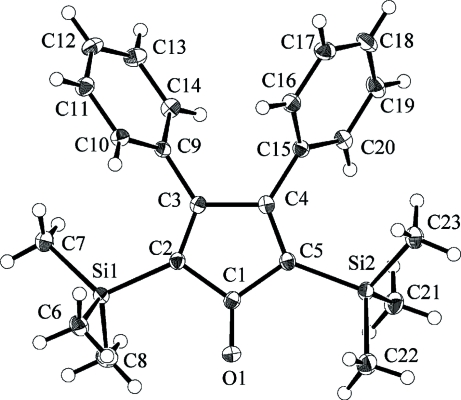
A view of the molecule of (I) showing the atom-labelling scheme. Displacement ellipsoids are drawn at the 40% probability level.

## supplementary crystallographic information

### Comment

Although the synthesis of the title compound, (I), was already reported (Rajesh & Periasamy, 1999), the reported NMR data were incorrect. Thus, we report herein the molecular structure of the title compound and revise its NMR data. The five-membered ring and four bonds derived from the each of carbon atoms in the five-membered ring are situated in a planar geometry, as was observed in the tetraphenylcyclopentadienone (Barnes *et al.*, 1991). Bond alternation of the C—C bonds in the five-membered ring of (I) is found, as was observed in other cyclopentadienones (Barnes *et al.*, 1991; Ruffani *et al.*, 2006). The C1—O1 distance (1.2139 (15) Å) in (I), is quite similar to that found in the tetraphenyl derivative (Barnes *et al.*, 1991).

### Experimental

To lithium (32 mg, 4.61 mmol) was added a diethyl ether (2.5 ml) solution of phenyl(trimethylsilyl)acetylene (408 mg, 2.34 mmol) and the resulting mixture was stirred at room temperature for 4 h. To an ether solution of 1,4-dilithio-1,3-butadiene thus obtained was added diethyl ether (8 ml) and unreacted lithium was removed by filtration. Carbon dioxide was bubbled into the filtrate at room temperature for 1 min. After treatment of the resulting mixture with hydrochloric acid (3 N), the organic layer was extracted with diethyl ether and dried over anhydrous magnesium sulfate. After removal of volatile substances, the residue was subjected to column chromatography (SiO_2_, hexane:ethyl acetate = 30:1) to afford the title compound, 3,4-diphenyl-2,5-bis(trimethylsilyl)cyclopentadienone (I) (22 mg, 5%). Suitable crystals for X-ray crystallographic analysis were obtained by slow evaporation of a hexane solution of (I).

### Refinement

Hydrogen atoms attached to C(*sp*^3^) and C(*sp*^2^) carbon atoms were treated as riding with C—H distances of 0.96 and 0.93 Å, respectively, and were included in the final cycles of least squares with isotropic Uijs by using a riding model, while all the other atoms were refined anisotropically.

### Crystal data

**Table:**

C_23_H_28_OSi_2_	*F*(000) = 808
*M**_r_* = 376.63	*D*_x_ = 1.132 Mg m^−^^3^
Orthorhombic, *P*2_1_2_1_2_1_	Mo *K*α radiation, λ = 0.71073 Å
Hall symbol: P 2ac 2ab	Cell parameters from 5798 reflections
*a* = 9.8418 (6) Å	θ = 2.7–27.9°
*b* = 11.8041 (7) Å	µ = 0.17 mm^−^^1^
*c* = 19.0181 (12) Å	*T* = 103 K
*V* = 2209.4 (2) Å^3^	Cube, orange
*Z* = 4	0.50 × 0.40 × 0.40 mm

### Data collection

**Table:**

Bruker SMART CCD area-detector diffractometer	5286 independent reflections
Radiation source: fine-focus sealed tube	5181 reflections with *I* > 2σ(*I*)
graphite	*R*_int_ = 0.021
φ and ω scans	θ_max_ = 27.9°, θ_min_ = 2.0°
Absorption correction: multi-scan (SADABS; Sheldrick, 1996)	*h* = −12→11
*T*_min_ = 0.922, *T*_max_ = 0.935	*k* = −15→15
16200 measured reflections	*l* = −21→25

### Refinement

**Table:**

Refinement on *F*^2^	Secondary atom site location: difference Fourier map
Least-squares matrix: full	Hydrogen site location: inferred from neighbouring sites
*R*[*F*^2^ > 2σ(*F*^2^)] = 0.028	H-atom parameters constrained
*wR*(*F*^2^) = 0.075	*w* = 1/[σ^2^(*F*_o_^2^) + (0.0473*P*)^2^ + 0.2508*P*] where *P* = (*F*_o_^2^ + 2*F*_c_^2^)/3
*S* = 1.07	(Δ/σ)_max_ = 0.001
5286 reflections	Δρ_max_ = 0.28 e Å^−^^3^
241 parameters	Δρ_min_ = −0.20 e Å^−^^3^
0 restraints	Absolute structure: Flack (1983), with 2292 Friedel pairs
Primary atom site location: structure-invariant direct methods	Flack parameter: 0.01 (6)

### Special details

**Table:**

Geometry. All e.s.d.'s (except the e.s.d. in the dihedral angle between two l.s. planes) are estimated using the full covariance matrix. The cell e.s.d.'s are taken into account individually in the estimation of e.s.d.'s in distances, angles and torsion angles; correlations between e.s.d.'s in cell parameters are only used when they are defined by crystal symmetry. An approximate (isotropic) treatment of cell e.s.d.'s is used for estimating e.s.d.'s involving l.s. planes.
Refinement. Refinement of *F*^2^ against ALL reflections. The weighted *R*-factor *wR* and goodness of fit *S* are based on *F*^2^, conventional *R*-factors *R* are based on *F*, with *F* set to zero for negative *F*^2^. The threshold expression of *F*^2^ > σ(*F*^2^) is used only for calculating *R*-factors(gt) *etc*. and is not relevant to the choice of reflections for refinement. *R*-factors based on *F*^2^ are statistically about twice as large as those based on *F*, and *R*- factors based on ALL data will be even larger. The Flack parameter was not changed after least-square refinement without merging the reflections. When the refinement was carried out using the opposite absolute structure, the Flack parameter was 0.99 (7).

### Fractional atomic coordinates and isotropic or equivalent isotropic displacement parameters (Å^2^)

**Table:**

	*x*	*y*	*z*	*U*_iso_*/*U*_eq_	
Si1	0.18748 (3)	1.12044 (3)	0.976841 (17)	0.01882 (8)	
Si2	0.42760 (3)	0.77489 (3)	0.787011 (18)	0.02008 (8)	
O1	0.42093 (9)	1.01676 (8)	0.87657 (5)	0.0272 (2)	
C2	0.18860 (12)	0.99471 (10)	0.91621 (6)	0.0180 (2)	
C15	0.08537 (12)	0.72378 (10)	0.82868 (6)	0.0190 (2)	
C7	0.03840 (14)	1.11986 (13)	1.03721 (7)	0.0289 (3)	
H7A	0.0361	1.0497	1.0627	0.043*	
H7B	−0.0436	1.1278	1.0103	0.043*	
H7C	0.0459	1.1818	1.0697	0.043*	
C3	0.09673 (12)	0.91490 (9)	0.89917 (6)	0.0176 (2)	
C6	0.18912 (15)	1.25143 (10)	0.92156 (7)	0.0267 (3)	
H6A	0.1116	1.2513	0.8909	0.040*	
H6B	0.2708	1.2533	0.8940	0.040*	
H6C	0.1858	1.3170	0.9514	0.040*	
C16	−0.03609 (13)	0.73727 (11)	0.79091 (7)	0.0229 (2)	
H16	−0.0694	0.8096	0.7819	0.028*	
C12	−0.31728 (13)	0.90660 (12)	0.96478 (7)	0.0263 (3)	
H12	−0.4070	0.9059	0.9802	0.032*	
C9	−0.04729 (11)	0.91027 (10)	0.92063 (6)	0.0181 (2)	
C10	−0.13363 (12)	1.00076 (11)	0.90568 (6)	0.0209 (2)	
H10	−0.1009	1.0628	0.8807	0.025*	
C14	−0.09904 (13)	0.81670 (11)	0.95650 (6)	0.0227 (2)	
H14	−0.0432	0.7548	0.9654	0.027*	
C4	0.16243 (12)	0.82381 (10)	0.85340 (6)	0.0181 (2)	
C18	−0.06037 (15)	0.53448 (11)	0.78188 (7)	0.0290 (3)	
H18	−0.1087	0.4716	0.7662	0.035*	
C8	0.34391 (14)	1.11229 (13)	1.03210 (7)	0.0318 (3)	
H8A	0.3455	1.1748	1.0644	0.048*	
H8B	0.4227	1.1154	1.0024	0.048*	
H8C	0.3440	1.0424	1.0580	0.048*	
C1	0.31734 (12)	0.96101 (10)	0.87823 (6)	0.0191 (2)	
C20	0.13106 (14)	0.61403 (11)	0.84330 (7)	0.0228 (2)	
H20	0.2109	0.6037	0.8686	0.027*	
C22	0.55418 (15)	0.87927 (12)	0.75313 (8)	0.0333 (3)	
H22A	0.6181	0.8412	0.7232	0.050*	
H22B	0.6015	0.9131	0.7919	0.050*	
H22C	0.5081	0.9371	0.7269	0.050*	
C13	−0.23314 (13)	0.81532 (12)	0.97897 (7)	0.0271 (3)	
H13	−0.2665	0.7532	1.0035	0.033*	
C11	−0.26813 (13)	0.99900 (12)	0.92774 (7)	0.0241 (3)	
H11	−0.3251	1.0597	0.9177	0.029*	
C5	0.29427 (12)	0.84939 (10)	0.84058 (6)	0.0185 (2)	
C23	0.34694 (14)	0.70279 (12)	0.71046 (7)	0.0292 (3)	
H23A	0.4161	0.6693	0.6816	0.044*	
H23B	0.2967	0.7571	0.6834	0.044*	
H23C	0.2865	0.6448	0.7270	0.044*	
C19	0.05826 (15)	0.52031 (11)	0.82031 (7)	0.0284 (3)	
H19	0.0891	0.4478	0.8307	0.034*	
C17	−0.10663 (13)	0.64309 (12)	0.76695 (7)	0.0275 (3)	
H17	−0.1855	0.6527	0.7407	0.033*	
C21	0.51771 (14)	0.67089 (12)	0.84432 (8)	0.0287 (3)	
H21A	0.4534	0.6176	0.8630	0.043*	
H21B	0.5612	0.7102	0.8823	0.043*	
H21C	0.5849	0.6313	0.8172	0.043*	

### Atomic displacement parameters (Å^2^)

**Table:**

	*U*^11^	*U*^22^	*U*^33^	*U*^12^	*U*^13^	*U*^23^
Si1	0.01810 (15)	0.01801 (15)	0.02036 (15)	−0.00020 (12)	−0.00082 (12)	−0.00287 (12)
Si2	0.02101 (15)	0.01866 (15)	0.02057 (15)	0.00210 (12)	0.00300 (12)	−0.00136 (12)
O1	0.0186 (4)	0.0265 (4)	0.0366 (5)	−0.0041 (4)	0.0035 (4)	−0.0071 (4)
C2	0.0175 (5)	0.0178 (5)	0.0188 (5)	0.0023 (4)	−0.0003 (4)	−0.0005 (4)
C15	0.0209 (5)	0.0197 (5)	0.0164 (5)	−0.0020 (5)	0.0017 (4)	−0.0011 (4)
C7	0.0289 (6)	0.0320 (7)	0.0257 (6)	−0.0020 (6)	0.0058 (5)	−0.0061 (5)
C3	0.0185 (5)	0.0167 (5)	0.0175 (5)	0.0021 (4)	−0.0003 (4)	0.0020 (4)
C6	0.0322 (6)	0.0188 (6)	0.0291 (6)	−0.0009 (5)	0.0004 (5)	−0.0004 (4)
C16	0.0236 (6)	0.0232 (6)	0.0220 (5)	−0.0004 (5)	−0.0011 (5)	−0.0002 (5)
C12	0.0178 (5)	0.0358 (7)	0.0253 (6)	−0.0019 (5)	0.0025 (5)	0.0021 (5)
C9	0.0171 (5)	0.0196 (5)	0.0175 (5)	−0.0005 (4)	−0.0025 (4)	−0.0023 (4)
C10	0.0200 (5)	0.0204 (5)	0.0224 (5)	−0.0003 (5)	−0.0006 (4)	0.0025 (4)
C14	0.0232 (6)	0.0230 (6)	0.0219 (6)	0.0017 (5)	0.0000 (5)	0.0042 (4)
C4	0.0216 (6)	0.0166 (5)	0.0162 (5)	0.0022 (4)	−0.0010 (4)	0.0015 (4)
C18	0.0361 (7)	0.0251 (6)	0.0256 (6)	−0.0110 (5)	0.0011 (6)	−0.0062 (5)
C8	0.0267 (6)	0.0374 (7)	0.0315 (7)	0.0009 (6)	−0.0092 (5)	−0.0060 (6)
C1	0.0180 (5)	0.0190 (5)	0.0202 (5)	0.0028 (4)	−0.0005 (4)	0.0008 (4)
C20	0.0265 (6)	0.0207 (6)	0.0211 (5)	−0.0003 (5)	−0.0004 (5)	−0.0003 (5)
C22	0.0341 (7)	0.0291 (6)	0.0367 (7)	−0.0043 (6)	0.0147 (6)	−0.0016 (6)
C13	0.0251 (6)	0.0316 (7)	0.0246 (6)	−0.0039 (5)	0.0021 (5)	0.0075 (5)
C11	0.0194 (6)	0.0264 (6)	0.0264 (6)	0.0035 (5)	−0.0020 (5)	0.0007 (5)
C5	0.0205 (6)	0.0167 (5)	0.0183 (5)	0.0015 (4)	−0.0007 (4)	0.0002 (4)
C23	0.0331 (7)	0.0327 (7)	0.0217 (6)	−0.0001 (5)	0.0027 (5)	−0.0064 (5)
C19	0.0383 (7)	0.0183 (6)	0.0285 (6)	−0.0024 (5)	0.0024 (6)	−0.0005 (5)
C17	0.0251 (6)	0.0340 (7)	0.0234 (6)	−0.0052 (5)	−0.0028 (5)	−0.0023 (5)
C21	0.0269 (6)	0.0269 (6)	0.0323 (7)	0.0074 (5)	−0.0012 (5)	0.0004 (5)

### Geometric parameters (Å, °)

**Table:**

Si1—C7	1.8630 (13)	C9—C14	1.3946 (17)
Si1—C8	1.8666 (14)	C10—C11	1.3887 (17)
Si1—C6	1.8699 (13)	C10—H10	0.9300
Si1—C2	1.8795 (12)	C14—C13	1.3873 (19)
Si2—C23	1.8640 (14)	C14—H14	0.9300
Si2—C21	1.8659 (14)	C4—C5	1.3544 (17)
Si2—C22	1.8668 (14)	C18—C19	1.388 (2)
Si2—C5	1.8796 (12)	C18—C17	1.390 (2)
O1—C1	1.2139 (15)	C18—H18	0.9300
C2—C3	1.3454 (16)	C8—H8A	0.9600
C2—C1	1.5117 (16)	C8—H8B	0.9600
C15—C20	1.3993 (17)	C8—H8C	0.9600
C15—C16	1.4037 (17)	C1—C5	1.5166 (16)
C15—C4	1.4799 (16)	C20—C19	1.3886 (18)
C7—H7A	0.9600	C20—H20	0.9300
C7—H7B	0.9600	C22—H22A	0.9600
C7—H7C	0.9600	C22—H22B	0.9600
C3—C9	1.4760 (16)	C22—H22C	0.9600
C3—C4	1.5271 (16)	C13—H13	0.9300
C6—H6A	0.9600	C11—H11	0.9300
C6—H6B	0.9600	C23—H23A	0.9600
C6—H6C	0.9600	C23—H23B	0.9600
C16—C17	1.3876 (18)	C23—H23C	0.9600
C16—H16	0.9300	C19—H19	0.9300
C12—C11	1.3855 (19)	C17—H17	0.9300
C12—C13	1.3855 (19)	C21—H21A	0.9600
C12—H12	0.9300	C21—H21B	0.9600
C9—C10	1.3942 (16)	C21—H21C	0.9600
			
C7—Si1—C8	107.60 (6)	C15—C4—C3	121.72 (10)
C7—Si1—C6	110.88 (7)	C19—C18—C17	119.63 (12)
C8—Si1—C6	110.62 (7)	C19—C18—H18	120.2
C7—Si1—C2	112.32 (6)	C17—C18—H18	120.2
C8—Si1—C2	107.45 (6)	Si1—C8—H8A	109.5
C6—Si1—C2	107.94 (5)	Si1—C8—H8B	109.5
C23—Si2—C21	110.99 (6)	H8A—C8—H8B	109.5
C23—Si2—C22	108.42 (7)	Si1—C8—H8C	109.5
C21—Si2—C22	108.58 (7)	H8A—C8—H8C	109.5
C23—Si2—C5	109.86 (6)	H8B—C8—H8C	109.5
C21—Si2—C5	108.84 (6)	O1—C1—C2	125.01 (11)
C22—Si2—C5	110.13 (6)	O1—C1—C5	125.76 (11)
C3—C2—C1	105.30 (10)	C2—C1—C5	109.19 (10)
C3—C2—Si1	134.17 (9)	C19—C20—C15	120.61 (12)
C1—C2—Si1	120.39 (8)	C19—C20—H20	119.7
C20—C15—C16	118.71 (11)	C15—C20—H20	119.7
C20—C15—C4	120.72 (11)	Si2—C22—H22A	109.5
C16—C15—C4	120.56 (11)	Si2—C22—H22B	109.5
Si1—C7—H7A	109.5	H22A—C22—H22B	109.5
Si1—C7—H7B	109.5	Si2—C22—H22C	109.5
H7A—C7—H7B	109.5	H22A—C22—H22C	109.5
Si1—C7—H7C	109.5	H22B—C22—H22C	109.5
H7A—C7—H7C	109.5	C12—C13—C14	119.96 (12)
H7B—C7—H7C	109.5	C12—C13—H13	120.0
C2—C3—C9	127.20 (11)	C14—C13—H13	120.0
C2—C3—C4	110.23 (10)	C12—C11—C10	119.88 (12)
C9—C3—C4	122.56 (10)	C12—C11—H11	120.1
Si1—C6—H6A	109.5	C10—C11—H11	120.1
Si1—C6—H6B	109.5	C4—C5—C1	104.61 (10)
H6A—C6—H6B	109.5	C4—C5—Si2	131.46 (9)
Si1—C6—H6C	109.5	C1—C5—Si2	123.91 (9)
H6A—C6—H6C	109.5	Si2—C23—H23A	109.5
H6B—C6—H6C	109.5	Si2—C23—H23B	109.5
C17—C16—C15	120.21 (11)	H23A—C23—H23B	109.5
C17—C16—H16	119.9	Si2—C23—H23C	109.5
C15—C16—H16	119.9	H23A—C23—H23C	109.5
C11—C12—C13	120.17 (12)	H23B—C23—H23C	109.5
C11—C12—H12	119.9	C18—C19—C20	120.27 (12)
C13—C12—H12	119.9	C18—C19—H19	119.9
C10—C9—C14	118.95 (11)	C20—C19—H19	119.9
C10—C9—C3	120.03 (11)	C16—C17—C18	120.54 (12)
C14—C9—C3	121.02 (11)	C16—C17—H17	119.7
C11—C10—C9	120.52 (12)	C18—C17—H17	119.7
C11—C10—H10	119.7	Si2—C21—H21A	109.5
C9—C10—H10	119.7	Si2—C21—H21B	109.5
C13—C14—C9	120.49 (12)	H21A—C21—H21B	109.5
C13—C14—H14	119.8	Si2—C21—H21C	109.5
C9—C14—H14	119.8	H21A—C21—H21C	109.5
C5—C4—C15	127.71 (11)	H21B—C21—H21C	109.5
C5—C4—C3	110.56 (10)		
